# The analysis of genetic structure and characteristics of the chloroplast genome in different Japanese apricot germplasm populations

**DOI:** 10.1186/s12870-022-03731-5

**Published:** 2022-07-21

**Authors:** Xiao Huang, Daouda Coulibaly, Wei Tan, Zhaojun Ni, Ting Shi, Hantao Li, Faisal Hayat, Zhihong Gao

**Affiliations:** College of Horticulture, Nanjing Agricultural University, Nanjing, 210095 Jiangsu China

**Keywords:** Japanese apricot, Chloroplast genome, Comparative genomics, Genetic diversity, Phylogenetic analysis

## Abstract

**Background:**

Chloroplast (cp) genomes are generally considered to be conservative and play an important role in population diversity analysis in plants, but the characteristics and diversity of the different germplasm populations in Japanese apricot are still not clear.

**Results:**

A total of 146 cp genomes from three groups of wild, domesticated, and bred accessions of Japanese apricot were sequenced in this study. The comparative genome analysis revealed that the 146 cp genomes were divided into 41 types, and ranged in size from 157,886 to 158,167 bp with a similar structure and composition to those of the genus *Prunus.* However, there were still minor differences in the cp genome that were mainly caused by the contraction and expansion of the IR region, and six types of SSR in which mono-nucleotide repeats were the most dominant type of repeats in the cp genome. The genes *rpl33* and *psbI*, and intergenic regions of *start-psbA*, *rps3-rpl22*, and *ccsA-ndhD*, showed the highest nucleotide polymorphism in the whole cp genome. A total of 325 SNPs were detected in the 146 cp genomes, and more than 70% of the SNPs were in region of large single-copy (LSC). The SNPs and haplotypes in the cp genome indicated that the wild group had higher genetic diversity than the domesticated and bred groups. In addition, among wild populations, Southwest China, including Yunnan, Tibet, and Bijie of Guizhou, had the highest genetic diversity. The genetic relationship of Japanese apricot germplasm resources in different regions showed a degree of correlation with their geographical distribution.

**Conclusion:**

Comparative analysis of chloroplast genomes of 146 Japanese apricot resources was performed to analyze the used to explore the genetic relationship and genetic diversity among Japanese apricot resources with different geographical distributions, providing some reference for the origin and evolution of Japanese apricot.

**Supplementary Information:**

The online version contains supplementary material available at 10.1186/s12870-022-03731-5.

## Introduction

Japanese apricot (*Prunus mume* Sieb. et Zucc.) is a deciduous tree of the Rosaceae family and *Prunus* genus. It originated from southwest China and has been widely cultivated throughout East Asia and Japan. fruit is rich in nutrition, contains a variety of biological active substances and organic acids, has the function of regulating intestines and stomach and promoting digestion, known as healthy food. With the development of science and technology and people’s needs, the processed products of Japanese apricot are becoming more and more diversified. Fresh Japanese apricot fruit and its processed products are rich in nutrients with high medicinal value, and have progressively become functional foods for people today [1–3]. Japanese apricot is native to China, has a wide wild distribution, and a long history of cultivation [4]. There are still wild Japanese apricot tree communities in a natural state in many remote mountainous areas in southern China. As a species in natural vegetation, the Japanese apricot plays a role in the process of dynamic succession. In recent years, scholars have investigated and studied this tree in the Hengduan Mountains and the Yunnan-Guizhou Plateau on the border of Sichuan, Guizhou, Yunnan, and Tibet in China, which are the natural distribution centers of Japanese apricot and are also the main places where Japanese apricot shows natural variations and are thus the centers of genetic diversity of Japanese apricot [5]. Wild Japanese apricot trees are distributed over a wide range in China, including the Yangtze River Basin, the Pearl River Basin, Southwest China, and Taiwan. A pattern of overall continuity and partial discontinuity occurs within the natural distribution range of Japanese apricot [4]. However, the genetic relationship and diversity of Japanese apricots in these different regions has not yet been clarified.

Chloroplast (CP) is an important organelle structure in green plants. It can convert solar energy into carbohydrates through photosynthesis, and is considered as the metabolic center to maintain life activities on earth. It is an organelle with semi-reserved replication characteristics and has independent genetic material [6]. The chloroplast genome is an ideal system for studying the development of the plant system. Compared with the nuclear genome, the chloroplast genome is much smaller, and the genome composition and sorting are conservative, which can maintain the homology of the species gene [7]. The chloroplast genome followed maternal unisexual inheritance to replicate and transmit genome information [8]. The nucleotide replacement rate is moderate and basically a single copy genome, which can ensure that the population inheritance is not disturbed by collateral genes to the greatest extent [9]. The nucleotide substitution rate of coding region fragments of chloroplast genome is low and the rate is slow, and so it is often used to compare higher taxa (family and genus level). In contrast the substitution rate of the non-coding region is high and evolution is fast, making it suitable for comparing lower taxa (interspecific or subspecific) and recently differentiated taxa [10]. Therefore, cp DNA is suitable for studying plant phylogeography, examining population genetic structure and historical population dynamics [11, 12]. Cp DNA sequence markers have been widely used to study intraspecific lineage differentiation and interspecific gene flow. Therefore, in the study of plant geographical origin and evolution, chloroplast genome, as an important genome for studying genetic relationship, gene flow and genetic situation, is an important data source for the phylogeny of higher plant comparative genomics [11, 13].

The main objective of this study was thus to sequence the cp genomes of 146 Japanese apricot resources with different germplasm populations, and to compare and analyze these cp genomes in terms of genome structure, gene number and function, repetitive sequences, and nucleotide variability. Meanwhile, SNP molecular markers of the 146 cp genome sequences were used to explore the genetic relationship and genetic diversity among Japanese apricot resources with different geographical distributions, providing some reference for the origin and evolution of Japanese apricot.

## Results

### General features of the chloroplast genome

**Fig. 1:**
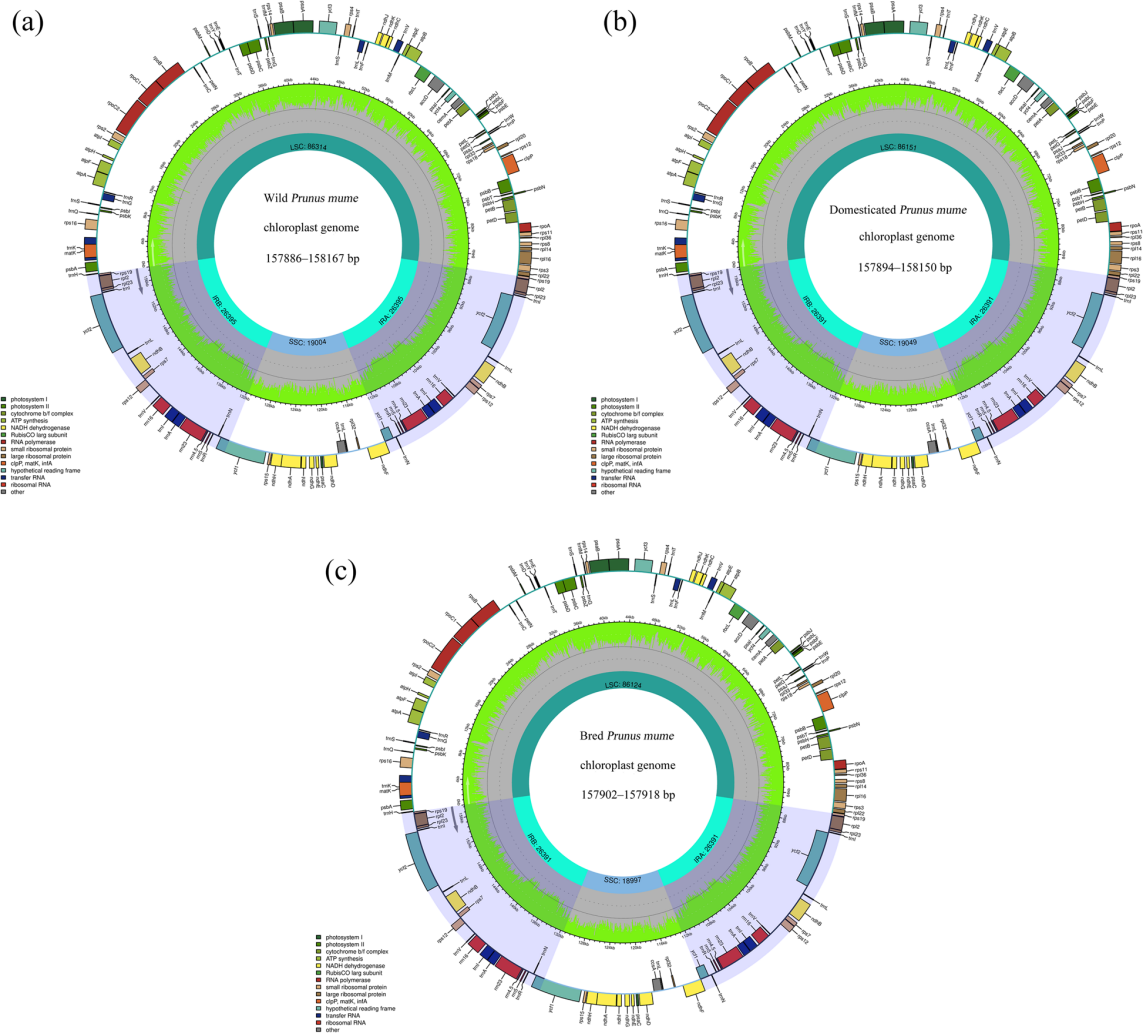
Chloroplast genome maps of Japanese apricot. **a** The chloroplast genome of wild group; **b** The chloroplast genome of domesticated group; **c** The chloroplast genome of bred group. Genes inside the circle are transcribed clockwise; genes outside are transcribed counter-clockwise. The dark grey inner circle corresponds to the AT content, the green to the GC content. Genes belonging to different functional groups are color-coded

The 146 cp genomes range in size from 157,886 to 158,167 bp. They are a typical ring structure, mainly composed of four parts, a pair of inverted repeat regions (26391–26,395 bp), a small single-copy region (18992–19,049 bp), and a large single-copy region (86084–86,379 bp). The average GC content of all cp genomes was 36.74%, the highest was 36.75%, the lowest was 36.72%, and the GC content of different regions also differed. The GC content was 34.53–34.58% in the LSC region, 30.22–30.42% in the SSC region and 42.56–42.58% in the IR region (Table S1). There are some differences in the length and GC content of chloroplast genome in different groups. In the wild group, the length of chloroplast genome ranges from 157,886 to 158,167 (Fig. 1a), LSC region ranges from 86,064 to 86,379, SSC region ranges from 18,992 to 19,049, and IR region ranges from 26,391 to 26,395. The total GC content ranges from 36.72 to 36.75, the GC content in LSC region ranges from 34.53 to 34.58, SSC region ranges from 30.32 to 30.42, and IR region ranges from 42.56 to 42.58. In the domesticated group, the length of chloroplast genome ranges from 157,894 to 158,150 (Fig. 1b), LSC region ranges from 86,107 to 86,348, SSC region ranges from 18,997 to 19,049, and IR region ranges from 26,391 to 26,395. The total GC content ranges from 36.72 to 36.75, the GC content in LSC region ranges from 34.55 to 34.58, SSC region ranges from 30.32 to 30.39, and IR region ranges from 42.56 to 42.57. In the bred group, the length of chloroplast genome ranges from 157,902 to 157,918 (Fig.1c), LSC region ranges from 86,114 to 86,125, SSC region ranges from 18,997 to 19,020, and IR region is 26,391. The total GC content is 36.74, the GC content in LSC region ranges from 34.56 to 34.58, SSC region ranges from 30.36 to 30.38, and IR region ranges from 42.56 to 42.57.

The number of genes encoded by 146 chloroplast genomes is the same with a total of 130 genes encoded, among which 112 are unique, namely 78 CDS genes, 4 rRNA genes, and 30 tRNA genes. Among these genes, ten CDS genes (*atpF*, *ndhA*, *ndhB*, *petB*, *petD*, *rpl2*, *rpl16*, *rpoC1*, *rps12*, and *rps16*) each contain one intron, six tRNA genes (*trnA-UGC*, *trnG-GCC*, *trnI-GAU*, *trnK-UUU*, *trnL-UAA*, and *trnV-UAC*) each contain one intron, and two CDS genes (*clpP* and *ycf3*) each contain two introns. Interestingly, the *rps12* gene is trans-spliced, with 5′ end exons in the LSC region and 3′ end exons and introns in the IR region. In addition, a pseudogene (*rps19*) is present in all cp genomes. These genes can be divided into three categories according to their functions (Table S2). The first type of function is mainly related to photosynthesis, with 43 unique genes; the second category of function is mainly related to cp automatic transcription and translation, with 59 unique genes; the third category has 10 unique genes, mainly involved in other biosynthetic genes and open reading frames with unknown functions.

### Repeat structure and SSR analysis of chloroplast genomes

**Fig. 2:**
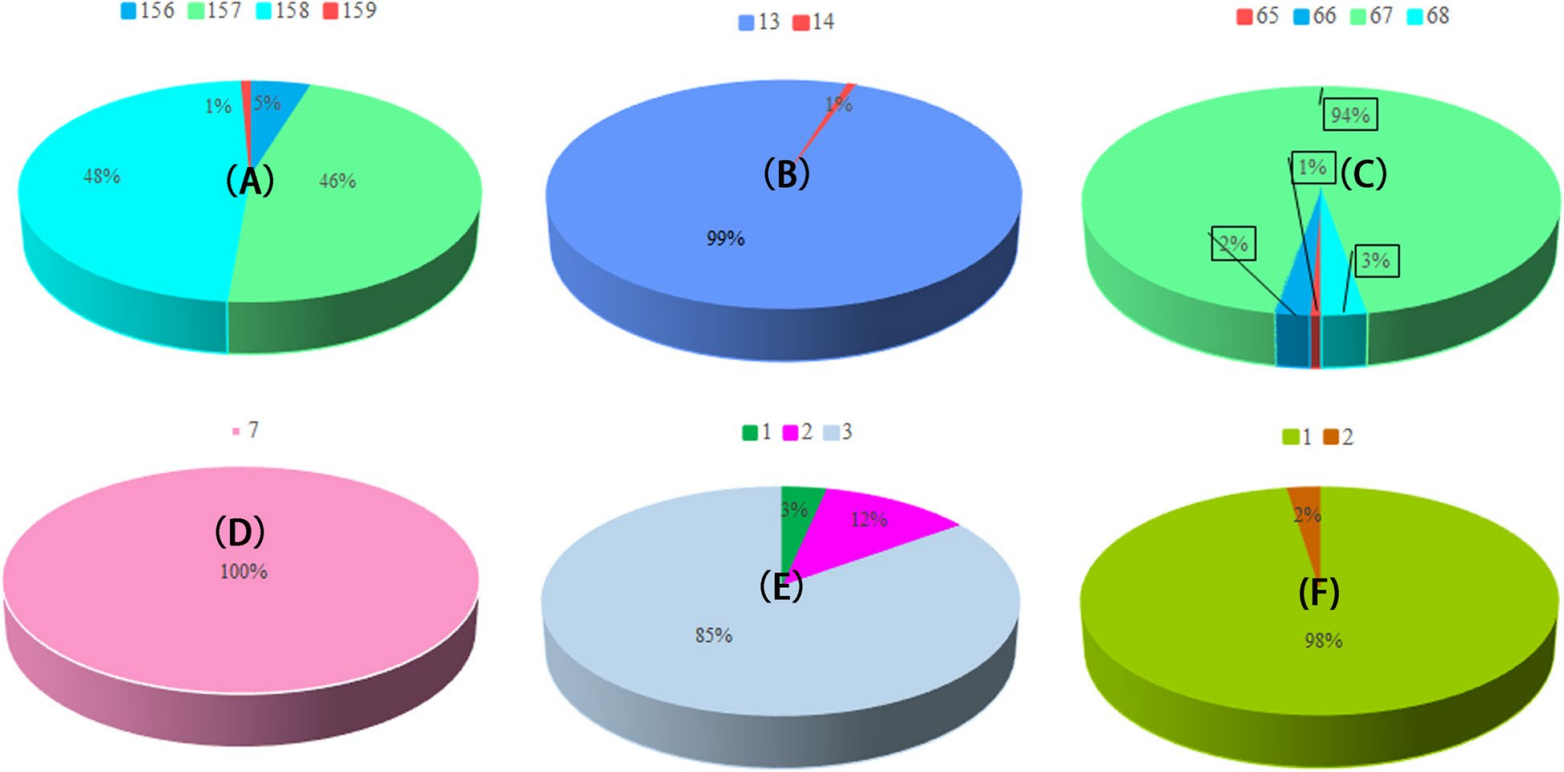
The types and numbers of SSRs in 146 Japanese apricot chloroplast genomes. **A** Mono-nucleotide repeats; **B** di-nucleotide repeats; **C** tri-nucleotide repeats; **D** tera-nucleotide repeats; **E** penta-nucleotide repeats; **F** hexa-nucleotide repeats

We detected six types of SSR (Fig. 2 and Table S3), in which mono-nucleotide repeats were the most dominant type of repeat in the cp genome. Among all the samples, the number of mono-nucleotide repeats fell into four groups: 5% of samples contained 156 repeats, 46% contained 157 repeats, 48% contained 158 repeats, and 1% contained 159 repeats. There were two types of di-nucleotide repeats in all samples, with 13 repeats in 99% of samples, and 14 repeats in the remaining 1%. There were four types of tri-nucleotide repeats: 65 repeats in 1% of samples, 66 repeats in 2% of samples, 67 repeats in 94% of samples, and 68 repeats in 3% of samples. There were 7 T-nucleotide repeats in all samples. There were three types of penta-nucleotide repeat: 3% of samples contained one repeat, 12% of samples contained two repeats, and 85% of samples contained three repeats. Hexa-nucleotide repeats were the least common type of repeat; 98% of samples with repeats contained one repeat, and 2% of samples with repeats contained two repeats.

**Fig. 3:**
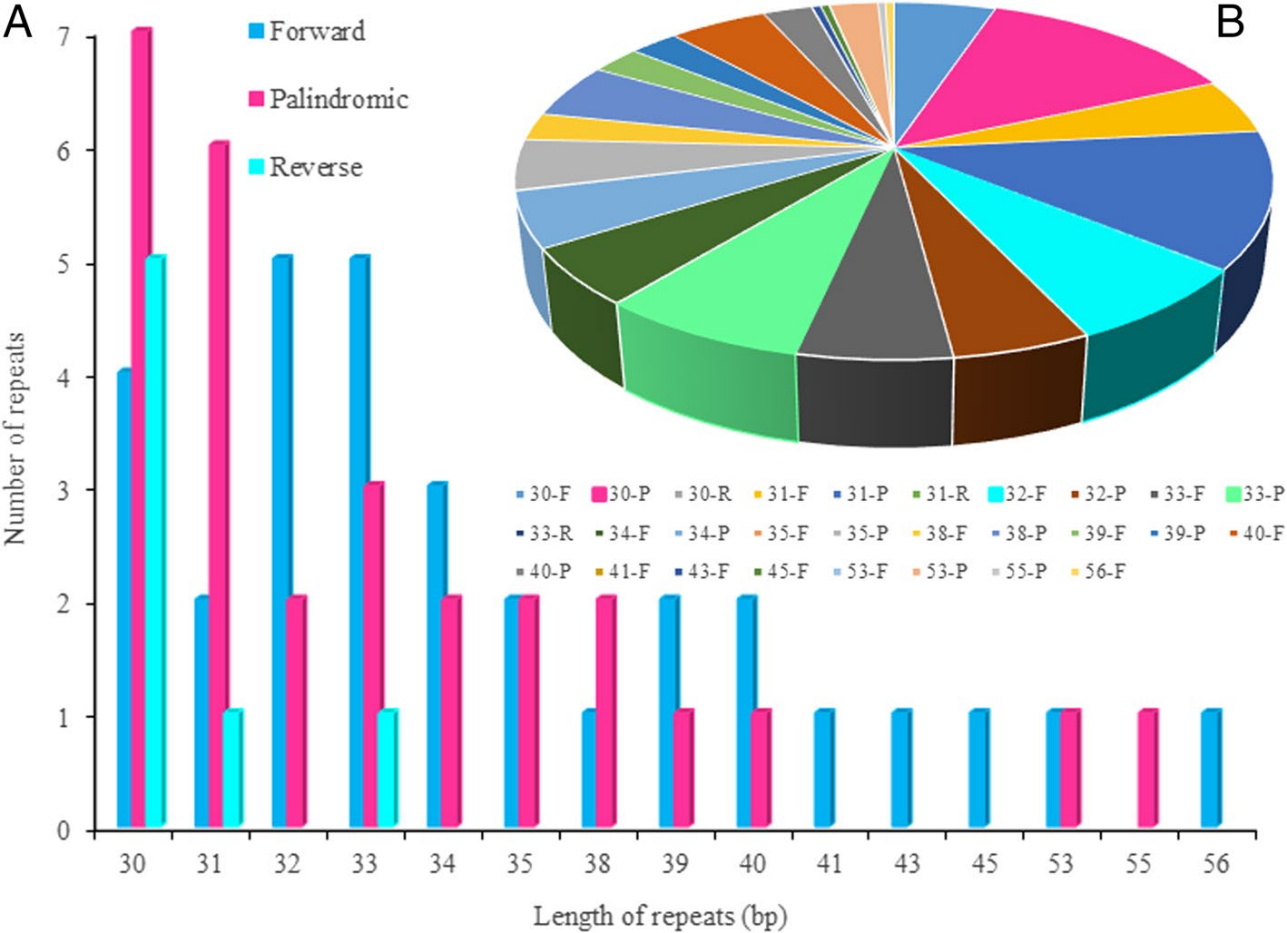
The repeats in 146 Japanese apricot chloroplast genomes. **A** Type and maximum number of repeats; **B** the proportion of all types of repeats; the number indicates the length of repeats, capital letters indicate the type of repeat (F: forward; P: palindromic; R: reverse)

Meanwhile, we found more than 30 bp of base repeats in all samples and distinct forms of repeats, such as forward, reverse, and palindromic repeats, and 18 types of repeats of different lengths (Fig. 3 and Table S4). The repeat sequence with a length of 30 bp was the most common type, accounting for 18.44% of all the repeats; forward repeats accounted for 5.16%, palindromic repeats accounted for 13.20%, and reverse repeats accounted for 0.08% of all repeats. There was a maximum of four forward repeats of length 30 bp, a maximum of seven forward repeats, and a maximum of five palindromes in each sample. Repeats with a length of 31 bp accounted for 17.30% of all repeats, there were up to two forward repeats in each sample, up to six palindromes, and up to one reverse repeats of length 31 bp. Repeats with a length of 32 bp accounted for 12.13% of all repeats, there were up to five forward repeats and up to two palindromic repeats of length 32 bp in each sample. Repeats with a length of 33 bp accounted for 13.08% of all repeats, there was a maximum of five forward repeats in each sample, three palindromic repeats, and one reverse repeat. The repeats containing 34 bp accounted for 10.17% of all repeats, and the number of forward repeats with 34 bp was at most three and there were at most two palindromic repeats. Repeats with a length of 35 bp accounted for 4.62% of all repeats, there were at most two forward repeats and at most two palindromic repeats with a length of 35 bp in each sample. The repeat sequence with a length of 38 bp accounted for 7.50% of all repeats, there were no more than one forward repeat and two palindromic repeats with a length of 38 bp. Repeats with 39 bp accounted for 5.03% of all repeats, there were at most two forward repeats of length 39 bp and one palindromic repeat in each sample. The repeat sequence with a length of 40 bp accounted for 7.49% of all repeats, there was a maximum of two forward repeats in each sample and a maximum of one palindromic repeat of length 40 bp. Repeats with lengths of 41, 43, 45, and 56 bp were only forward repeats, accounting for 0.034, 0.44, 0.428, and 0.44% of all repeats, respectively. Repeat sequences with a length of 53 bp accounted for 2.50% of all repeats. In each sample, there was up to one forward repeats with a length of 53 bp and up to one palindromic repeat. Repeats with 55 bp were palindromic, accounting for 0.40% of all repeats.

### Comparative chloroplast genome analysis

The non-synonymous (K_A_) and synonymous (K_S_) nucleotide substitutions play an important role in plant evolution. The present study analyzed the substitution rates of K_A_/K_S_ in all protein-coding genes between *Prunus persica* and Japanese apricot (Table S5). In most Japanese apricots, the *ndhI*, *ccsA*, *ndhF*, *rpl22*, *rbcL*, *matK*, and *rpoC2* genes had the highest K_A_/K_S_ ratios (Fig. 5b). Among these, most *ndhI*, *ccsA*, and *ndhF* genes had K_A_/K_S_ ratios greater than 1, which indicated that these genes were positively selected. These are photosystem genes and are also important functional genes in the cp genome. In this study, the samples of Japanese apricot were collected from different geographical locations. The conditions of climate, water, light, and heat in the growth environment thus differed, which may be the main reason for positively selecting these genes. Therefore, we further analyzed these genes in wild samples from different geographical locations. The results (Table S5) show that the K_A_/K_S_ value of the *ndhF* gene in most wild samples was greater than 1. The K_A_/K_S_ values of *ndhI* and *ccsA* genes were related to their geographical location. The K_A_/K_S_ value of the *ccsA* gene in Guizhou, Zhejiang, Fujian, and Guangdong was greater than 1, whereas the K_A_/K_S_ value of the *ccsA* gene in Tibet and Yunnan was less than 1. The K_A_/K_S_ value of the *ndhI* gene in Guizhou, Zhejiang, Tibet, and Yunnan was greater than 1, while the K_A_/K_S_ value of the *ndhI* gene in Fujian and Guangdong was less than 1.

**Fig. 4:**
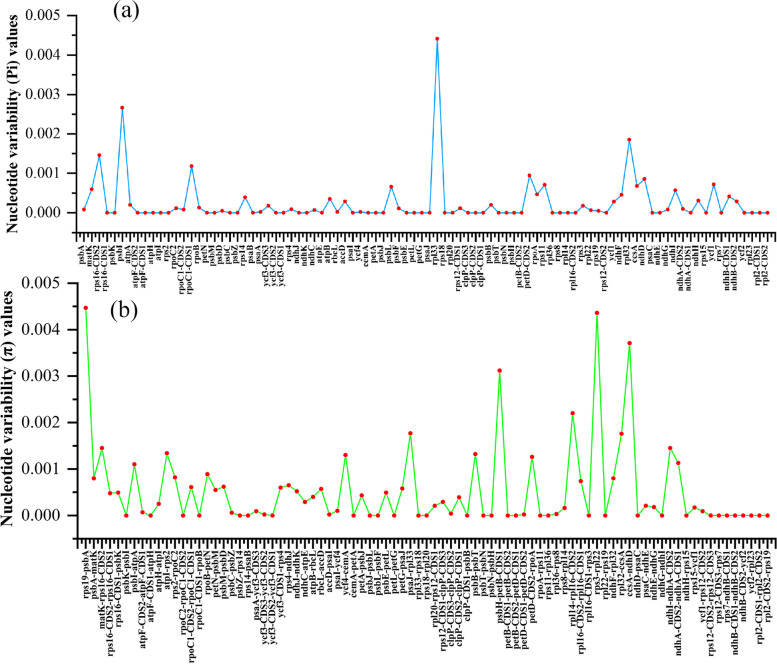
Comparison of the nucleotide variability (π) values among the 146 cp genomes. **a** CDS region, (**b**) intergenic region

Meanwhile, genes from all coding regions of 146 cp genomes were extracted and evaluated to analyze nucleotide variability. We found that there was nucleotide diversity in 42 different protein-coding genes, and that high nucleotide diversity was mainly distributed in LSC regions, rather than SSC and IR regions. The *rpl33*，*psbI*，*rpl32*，*rps16-CDS2*, *rpoC1-CDS1*, *petD-CDS2*, *ndhD*, *ycf1*, and *rps11* genes show the highest π values; among these, *rps33* showed the highest nucleotide variability (Fig. 4a). We also analyzed the nucleotide variability in all the intergenomic regions of the 146 cp genomes, and found that 52 intergenomic regions had nucleotide diversity. Compared with the coding region, the intergenic region showed higher nucleotide diversity, within which *rps19*-*psbA*, *rps3-rpl22*, *ccsA-ndhD*, *psbH-petB*, *rpl14-rpl16*, *psaJ-rpl33*, *rpl32-ccsA*, *matK-rps16*, and *ndhI-ndhA* had the highest π values, and *rps19*-*psbA* showed the highest nucleotide diversity (Fig. 4b).

We also evaluated codon information and codon usage frequency of 146 Japanese apricots chloroplast genomes. All protein-coding genes were encoded by 22,722 to 22,731 codons, the AUU-encoded isoleucine was the most frequent amino acid, and its frequency of use was 977 times. And the GUG- and UUG-encoded methionine was the least frequent amino acid and had only one time in most of the 146 Japanese apricots (Table S6). In these Japanese apricots, we found that the AUG-encoded methionine had the highest RSCU value at approximately 2.989, and the UUG-encoded methionine had the lowest RSCU value at approximately 0.0057 (Table S7).

### Population structure and phylogenetic tree analysis of chloroplast genomes

**Fig. 5:**
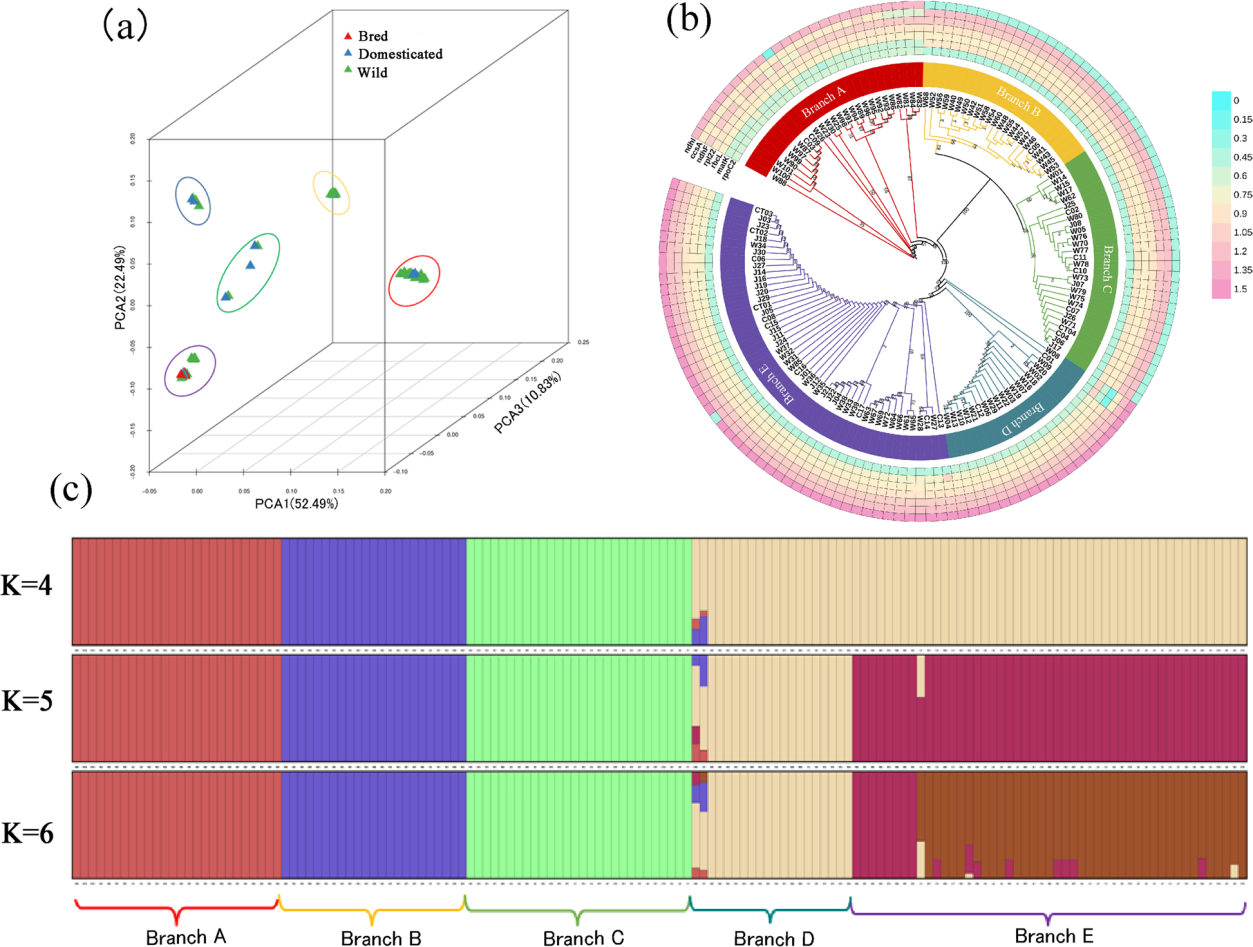
Genetic structure of 146 Japanese apricot chloroplast genomes. **a** Principal component analysis (PCA). **b** The phylogenetic tree was established by SNPs based on maximum likelihood. The inner circle represents the phylogenetic tree constructed based on SNPs of 146 chloroplast genomes, and the outer circle is the K_A_/K_S_ values heatmap of gene *rpoC2*, *matK*, *rbcL*, *rpl22*, *ndhF*, *ccsA*, and *ndhI* between *Prunus mume* and *Prunus persica*. **c** Population structure analysis of the 146 Japanese apricot accessions. Branch A to E represented the clustering information of population structure results, which corresponds to the phylogenetic tree

The results of principal component analysis (PCA) showed that PCA1, PCA2, and PCA3 represented 52.49, 22.49, and 10.83% of all variation, respectively (Fig. 5a). Among the groups, wild accessions from Tibet, Bijie of Guizhou, and Yunnan formed a closely related cluster, and a single group composed of wild samples from Libo of Guizhou. The samples from Japan, Guangdong, Taiwan, and Zhejiang formed a closely related cluster, and Fujian wild samples formed a closely related group. The results of phylogenetic tree showed that 146 germplasm resources were divided into 5 branches (Fig. 5b). Branch A comprised Tibet, Bijie of Guizhou, Yunnan wild samples and Jiangsu domesticated varieties. Branch B included Fujian wild germplasms and one domesticated variety from Sichuan. Branch C included Guizhou and Guangdong wild samples, some Japanese bred varieties and domesticated varieties from Fujian and Sichuan. Branch D included Libo of Guizhou wild accessions and some domesticated varieties from Hunan, Guizhou, and Yunnan. Branch E contained domesticated varieties from Taiwan, Zhejiang, and Guangdong, Fujian and Zhejiang wild germplasms, and most of the bred varieties.

The population structure analysis showed that when K = 4, wild germplasms from Bijie of Guizhou, Yunnan, Tibet and domesticated varieties in Jiangsu formed the first category; wild accessions from Fujian and domesticated varieties from Sichuan constituted the second-largest category; wild accessions from Guangdong, Libo, and Fujian, domesticated varieties from Fujian and Sichuan, and Japanese bred varieties formed the third category; wild accessions from Libo, Fujian, and Zhejiang, domesticated varieties from Taiwan, Guangdong, Zhejiang, Yunnan, Guizhou, and Hunan, and Japanese bred varieties formed the fourth largest category (Fig. 5c). When K = 5, the fourth category formed two new categories: the wild accessions from Libo and domesticated varieties from Yunnan and Guizhou formed one category, and the wild germplasms from Fujian and Zhejiang, domesticated varieties from Taiwan, Zhejiang, Guangdong, and Hunan, and Japanese bred varieties formed the other category. When K = 6, the fifth category formed two new categories: the wild samples from Fujian formed one category, and the wild samples from Zhejiang, domesticated varieties from Taiwan, Zhejiang, Guangdong, and Hunan, and Japanese bred varieties formed the other category. We found that when K = 5, the model that divided the genetic composition of the ancestral germplasm of the population into five categories best reflected the population genetic structure of the 146 accessions, which was consistent with the PCA and phylogenetic tree results.

### Genetic diversity and haplotype analysis in different germplasm populations

**Fig. 6:**
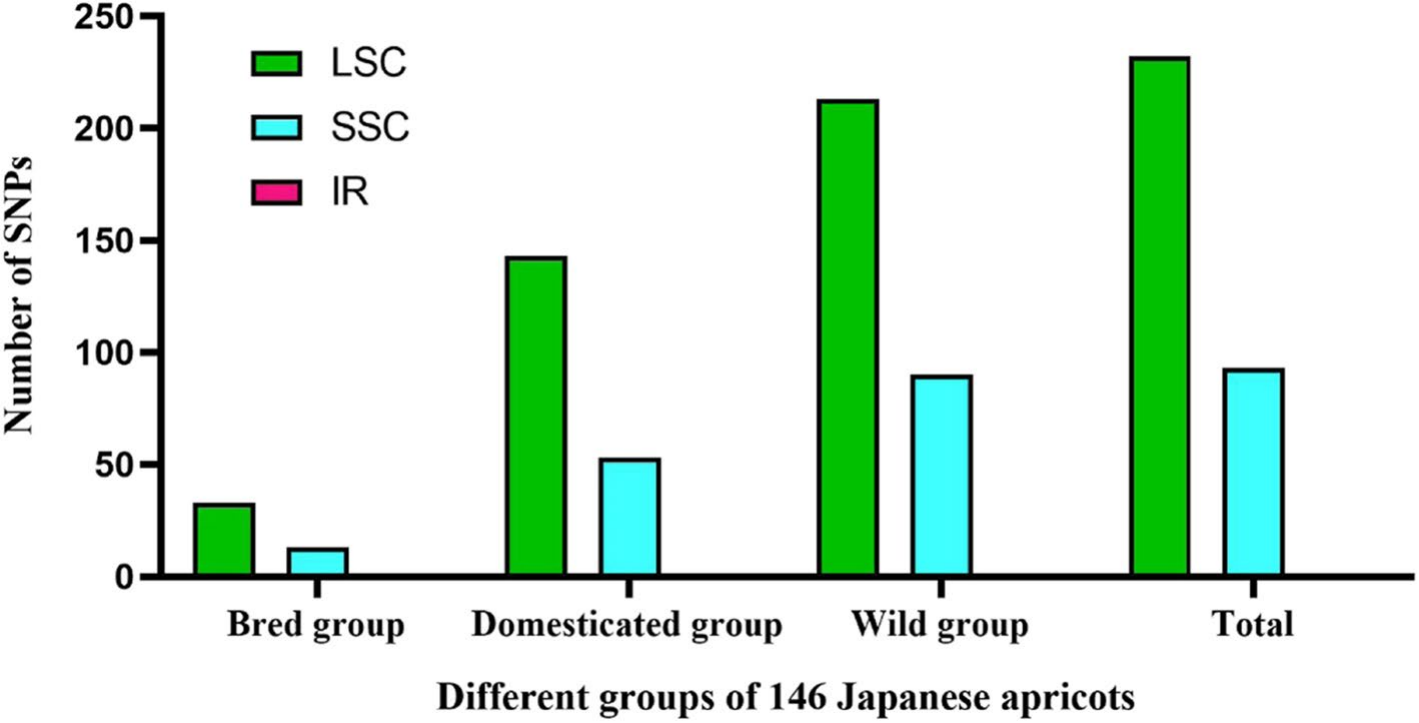
Distribution position and number of SNPs in different groups of 146 chloroplast genomes

A total of 325 SNPs were identified in 146 cp genomes. To further verify the accuracy of these SNPs, we randomly selected some sequences for verification by PCR. The results showed that the detection of SNPs was reliable (Fig. S1, S2, S3, S4 and S5). SNPs in different groups were mainly distributed in the LSC and SSC regions. The IR region was relatively conservative and no SNP was detected (Fig. 6). In addition, SNPs identified in the LSC region accounted for more than 70% of all SNPs in each group.

At the genome-wide level there were 46 SNPs accounting for 14.15% of all SNPs in the bred accessions, 196 SNPs accounting for 60.31% of all SNPs in the domesticated accessions, and 303 SNPs accounting for 93.23% of all SNPs in the wild germplasms (Table 1). In the genic region, there were 18 SNPs accounting for 13.63% of all SNPs in the bred accessions, 69 SNPs accounting for 52.27% of all SNPs in the domesticated accessions, and 128 SNPs accounting for 96.97% of all SNPs in the wild accessions (Table 2). The largest number of SNPs was identified in the wild accessions group, proving that the wild resource has a highly diversified gene pool and are valuable genetic improvement resources. However, at the genome-wide level, the total nucleotide diversity according to the π value in the 146 cp genomes was 0.2981 × 10^− 3^; the π of the wild group was 0.3250 × 10^− 3^, while the π values of the bred accessions and domesticated accessions were 0.2503 × 10^− 3^ and 0.1057 × 10^− 3^, which were significantly lower than that of the wild accessions group. The nucleotide diversity of the genic region showed similar results, indicating that the wild group had higher genetic diversity.

**Table 1:** Analysis of chloroplast genome-wide genetic diversity in different groups

Genome-wide	SNP number	π (10^−3^)	θW (10^− 3^)	Tajiama’s D
Bred group	46	0.1057	0.065	1.4195
Domesticated group	196	0.2503	0.2884	−1.1288
Wild group	303	0.325	0.3267	−0.4154
Total	325	0.2981	0.3292	−0.638

**Table 2:** Analysis of chloroplast genic region genetic diversity in different groups

Genic region	SNP number	π (10^−3^)	θW (10^−3^)	Tajiama’s D	Non-synonymous	Synonymous	Non-syn/syn
Bred group	18	0.0409	0.0254	1.2745	9	7	1.2857
Domesticated group	69	0.0922	0.1015	−0.9693	25	36	0.6944
Wild group	128	0.1238	0.138	−0.6935	55	59	0.9322
Total	132	0.1129	0.1337	−0.801	57	61	0.9344

**Fig. 7:**
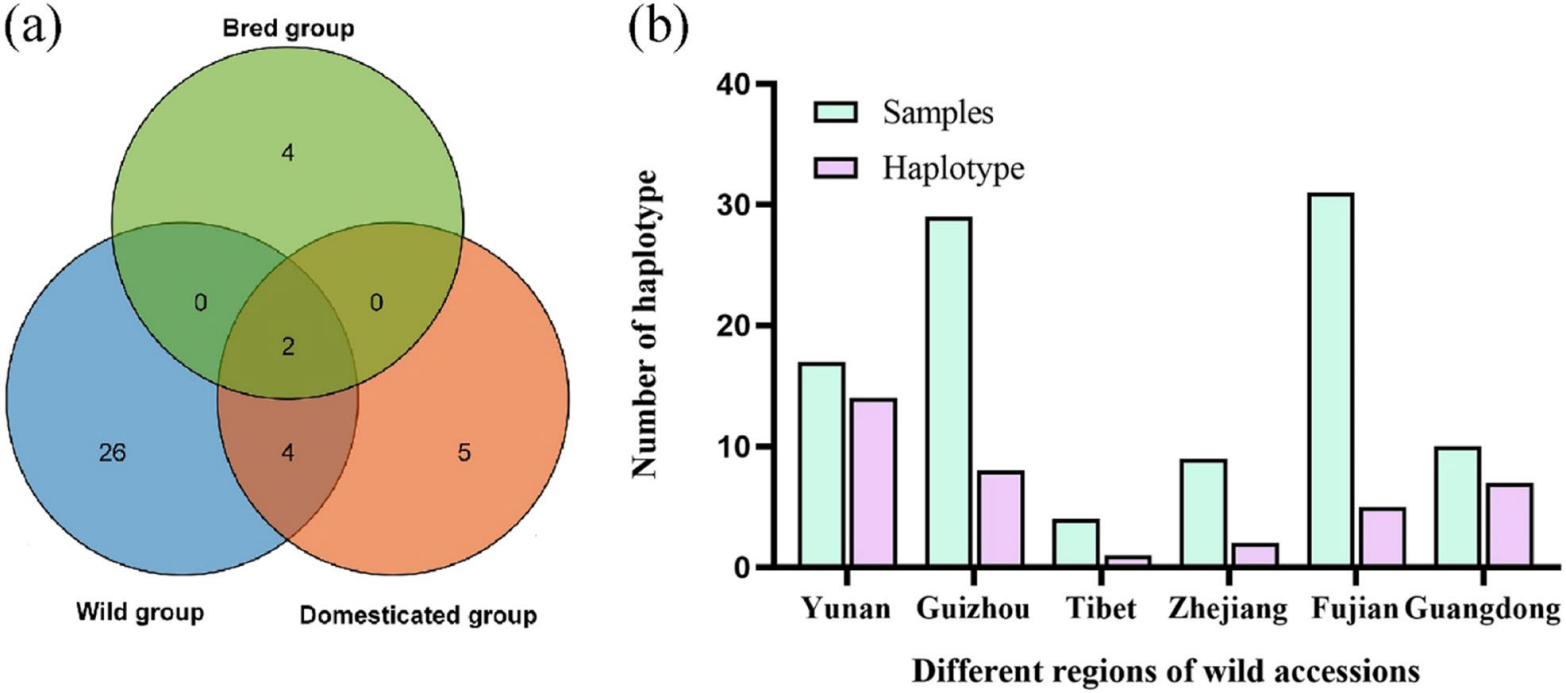
Haplotype analysis in different groups and regions. **a** Venn diagrams of the number of haplotypes in different groups. **b** Haplotype analysis of wild groups in different regions

Meanwhile, we also detected the haplotypes in chloroplast genome, and a total of 41 haplotypes were found in 146 samples (Table S8), among them, 32 haplotypes were identified in the wild group, accounting for 78.05% of all haplotypes and 26 were unique to the wild group. 11 haplotypes were identified in the domesticated group, accounting for 26.83% of all haplotypes and 5 of them were unique. 6 haplotypes were identified in the bred group, accounting for 14.63% of all haplotypes and 4 of them were unique. The three groups shared two haplotypes (Fig. 7a). The number of haplotypes in wild group was significantly higher than that in domesticated group and bred group. To further explore the haplotype information of wild populations, we analyzed the haplotypes of 100 samples from 7 different regions of wild populations. The results showed that there were 32 haplotypes in the wild population, of which the number of haplotypes in Yunnan region was the highest, with 14 haplotypes identified, accounting for 43.75% of the total number, followed by Guizhou region, with 8 haplotypes identified, accounting for 25% of the total number. The number of haplotypes identified in Tibet region is the least, only one, but the number of samples in Tibet is also the least. In addition, the proportion of haplotypes in Guangdong region is also very high (Fig. 7b).

## Discussion

In the present study, a total of 146 cp genomes were sequenced, and the size of the cp genomes ranged from 157,886 to 158,167 bp. The main reason for the difference in the size of the cp genomes was the large difference in the length of the LSC region. The SSR analysis revealed that mono-nucleotide repeats were the most common type of repeat in the cp genome, as found in several other species, including *Lilium* [14], *Allium* [15], and *Primula* [16]. These repeats are widely used as molecular markers in evolutionary studies and population genetics, and compared with other types of single nucleotide SSRs, most mono-nucleotide SSRs (A/T) were more abundant in the cp genome. These findings are consistent with previous studies of angiosperm cp genomes [17, 18]. It has been reported that this may be due to the ease of A-T conversion compared with G-C in the plant cp genome. Meanwhile, we detected three types of repeats (forward repeats, reverse repeats, and palindromic repeats), among which palindromic repeats were the most common type of repeat. Codon usage analysis is essential to understand the evolutionary process, genome structure and selection pressure on genes [19]. In the current study, the high level of similarities in codon usage revealed that the 146 Japanese apricots might have encountered similar environmental factors in the process of evolution. The results of RSCU in the 146 Japanese apricots showed that G and C were biased toward a lower nucleotide frequency than A and T at the third codon position, consistent with research on other angiosperm cp genomes [17].

The non-synonymous (K_A_) and synonymous (K_S_) patterns of nucleotide substitution are important signs in evolution [20]. The K_A_/K_S_ ratio indicates the selection pressure on genes. A K_A_/K_S_ value less than 1 indicates purification selection, equal to 1 indicates neutral evolution, and greater than 1 indicates positive selection [21]. In the present study, we observed low K_A_/K_S_ ratios (< 0.5) in most genes, indicating that purification selection is acting on these genes. However, *ndhI*, *ccsA*, and *ndhF* had higher K_A_/K_S_ values. The functions of these genes are mainly related to subunits of NADH dehydrogenase and cytochrome synthesis. They may be under large positive selection due to certain environmental conditions. To identify the sequence divergence hotspots, we analyzed the nucleotide diversity (π) value of the CDS region and intergenic region. Most sequence variations were found in the LSC and SSC regions, with high nucleotide variability in the LSC region, while the IR regions contained fewer sequence variations. The SNP analysis results also show that the LSC region has the most SNPs, accounting for more than 70% of all SNPs in the whole chloroplast genome. The nucleotide diversity (π) values in the intergenic region were higher than in the CDS region, which was consistent with previous studies [22, 23]. There were two CDS genes (*rpl33* and *psbI*) and five intergenomic regions (*rps19*-*psbA*, *rps3-rpl22*, *ccsA-ndhD*, *psbH-petB*, and *rpl14-rpl16*) that showed high nucleotide diversity values (π > 0.002). Together, these divergent hotspots could contribute to the development of molecular markers for phylogenetic analysis and the identification of *Prunus* species [24].

Japanese apricot originated in China and has been cultivated for more than 7000 years. The wild distribution center of Japanese apricot is generally believed to be in the Hengduan Mountains and Yunnan-Guizhou Plateau region of China [5]. The distribution of wild Japanese apricot is affected by the climatic conditions and soil environment in the growth area, resulting in natural selection [25]. The distribution center regions have undulating terrain, changeable climate, and diverse soil types, which support the plasticity of phenotypic and genetic diversity of Japanese apricot and lead to germplasm diversity [25, 26]. The results showed that the genetic diversity of wild populations was significantly higher than that of domesticated and bred groups, and the results of haplotype analysis also support this finding. The PCA, phylogenetic tree and population structure results showed that the Japanese apricots from Yunnan, Bijie of Guizhou, and Tibet were gathered, and were closely related. Haplotype analysis of wild populations also found higher diversity in southwest China, including Yunnan, Guizhou, and Tibet regions, which was basically consistent with our previous research results [27]. Southwest China is the origin center of Japanese apricot, and due to its superior geographical environment and unique cultural customs, wild Japanese apricot has developed well in this region. It also provides valuable wild resources for genetic improvement and breeding of Japanese apricot, and enriches the germplasm gene bank of Japanese apricot. The phylogenetic tree results showed that most of the samples from the Fujian and Guangdong regions were clustered together and were closely related; these two regions located in southern China and are geographically contiguous. In addition, the samples from Zhejiang and Japan were clustered together, being genetically closely related. Japanese apricot is distributed widely in Japan, which lie on a similar latitude to that of Jiangsu and Zhejiang [28]. The results of genetic diversity analysis of Japanese germplasm resources by REMAP and IRAP molecular markers showed that Japanese accessions were introduced from Zhejiang Province of China [29]. Therefore, we speculated that the genetic relationship of Japanese apricot germplasm resources in the different regions showed a degree of correlation with their geographical distribution. In addition, chloroplast genome can be used as an important data source to play a more and more important role in the study of genetic and evolutionary relationships of Japanese apricot. As the origin of Japanese apricot, China is also the country with the widest area suitable for Japanese apricot production in the world. Japanese apricot cultivation is available in many provinces throughout the country. In the future, we need to collect more germplasm resources to reveal the genetic and evolutionary relationship of Japanese apricot in these regions. Meanwhile, we will also explore the mechanism of dissemination and evolution of Japanese apricot in these regions, excavate some unique gene resources, and promote the development of Japanese apricot industry.

## Conclusion

In this study, a total of 146 cp genomes from different geographical locations were sequenced, and ranged in size from 157,886 to 158,167 bp with a similar structure and composition to the cp genomes of the genus *Prunus.* The comparative genome analysis revealed that the differences in the cp genomes were mainly caused by the contraction and expansion of the IR region. The *ndhI*, *ccsA*, and *ndhF* genes had a high K_A_/K_S_ ratio, and the *rpl33* and *psbI* genes and intergenic region of *rps19-psbA*, *rps3-rpl22*, and *ccsA-ndhD* showed the highest nucleotide diversity. A total of 325 SNPs were identified in 146 cp genomes, and more than 70% of the SNPs were in the LSC region. The genetic diversity and haplotype numbers of the wild group were significantly higher than these of domesticated and bred accessions groups. In addition, among wild populations, Southwest China has the highest genetic diversity. The study provided abundant chloroplast genome resources and made great contributions to the genetic diversity of Japanese apricot.

## Materials and methods

### Plant material

In this study, a total of 146 samples of Japanese apricot were collected from different regions, and were defined as the ‘wild group’ (100 samples), the ‘domesticated group’ (21 samples), and the ‘bred group’ (25 samples) (Table S9). All samples were identified by Prof. Zhihong Gao according to the flora of China, and they were kept in the National Field Gene Bank for *Prunus mume*, Nanjing, Jiangsu, China.

### Chloroplast sequencing and genome annotation

We used a modified CTAB method to extract DNA [30]. The sequencing library was constructed and PCR amplification was performed for quality inspection. After that the qualified library was sequenced using the Illumina Novaseq platform and the pairwise sequencing (PE) read length was 150 bp. The fastp (version 0.20.0, https://github.com/OpenGene/fastp) software was used to filter the original data and obtained the clean data. 146 Japanese apricot accessions generated 1576.80 Gb of clean data, the average sequencing data volume of each sample was 10.80 Gb, and the average sequencing depth was 46.14×. The sequencing data was of high quality (Q30 > 84.52%, Q20 > 92.69%). To reduce the complexity of subsequent sequence assembly, the software bowtie2 v2.2.4 (http://bowtie-bio.sourceforge.net/bowtie2/index.shtml) was used to compare the chloroplast genome database built by the company and chloroplast reference genome of *Prunus mume* [13], and the compared sequencing sequences were regarded as the chloroplast genome sequencing sequences of project samples. SPAdes [31] software is used to assemble the core module, and cp DNA sequence is assembled by SPAdes software to obtain seed sequence of chloroplast genome. Kmer iterative extend seed, if the result is a contig, the result is determined as pseudo genome sequence. The sequenced sequence is aligned to pseudo genome for genome correction. Otherwise, SSPACE V2.0 (https://www.baseclear.com/services/bioinformatics/basetools/sspace-standard/) software is used to connect the previously obtained contig sequences to obtain scaffolds; Gapfiller V2.1.1 (https://sourceforge.net/projects/gapfiller/) software was used to fill in the scaffolds sequence. If there was still gap after the above operation, primers were designed for PCR sequencing and assembly until a complete pseudo genome sequence was obtained, and then we aligned the sequenced sequence to the pseudo genome to determine the accuracy of the final assembly results for genome correction. Finally, according to the structure of chloroplast, the corrected pseudo genome was rearranged to obtain a complete chloroplast circular genome sequence. We compared the CDS sequences of the cp genome in NCBI by BLAST [32] software to obtain the annotation results. Hmmer [33] software was used to obtain rRNA annotation results of the cp genome sequence. The tRNA prediction of cp genome sequence was performed using Aragorn [34] software. The Chloroplot [35] software was used to draw the circular gene map of chloroplast genomes of Japanese apricot.

### Characterization of SSRs and repeat sequences

Simple sequence repeats (SSRs) were detected using the Perl script MISA [36], and the software REPuter [37] was used to visualize the location and size of the dispersed repeats (forward, reverse, and palindromic repeat sequences) with a minimum repeat size of 30 bp and a hamming distance of 3. The nucleotide diversity (π) values of Japanese apricot were evaluated and the regions of CDS and intergenic distances were calculated in DnaSP 5.1 [38].

### Codon usage analysis

The cp genome of 146 Japanese apricots was analyzed for the relative synonymous codon usage (RSCU). When the RSCU value > 1.00, codons were used more often than expected, and vice versa. The RSCU was determined using the software DAMBE5 [39].

### Variant calling and annotation

We used Bowtie software [40] to align the reference chloroplast genome with the reads of clean data. SAMtools [41] was used for variant calling, and VCFtools [42] was used to filter SNPs with a minor allele count higher than 3, a missing rate lower than 50%, and a minor allele frequency higher than 0.05. Subsequently, the ANNOVAR [43] software was used to evaluate the effect of variants.

### Phylogenetic tree, population structure, PCA, and haplotype analysis

The maximum likelihood phylogenetic tree was constructed based on filtered SNPs by RAxML software (raxml HPC-PTHREADS, version 8.2.12) [44], to analyze the genetic relationship of Japanese apricots in different regions. The population structure analysis was performed by admixture software (1.3.0) [45] and the PCA analysis was used by GCTA64 software (1.93.2) [46]. The 146 sequences were adjusted with the *psbA* gene as the starting point, and MAFFT (V7.427) was used for sequence alignment using default parameters [47]. We used DnaSP 5.1 software to detect chloroplast genome haplotypes [38].

## Supplementary Information

**Additional file 1.**

**Additional file 2.**

## Data Availability

The sequence data of Japanese apricot chloroplast genomes involved in this study have been deposited in GenBank (Accession No. BankIt2438332: MW755825–MW755870 and BankIt2436246: MW755871-MW755970). All relevant data can be found within the manuscript and its supporting materials.

## Abbreviations

CP: Chloroplast genome
SSR: Simple sequence repeat
IR: Inverted repeat
LSC: Large single copy
SSC: Small single copy
SNP: Single nucleotide polymorphism
PCA: Principal component analysis
PCR: Polymerase chain reaction
