# Supplementary material for: The analysis of genetic structure and characteristics of the chloroplast genome in different Japanese apricot germplasm populations
Source: BMC Plant Biol. 2022 Jul 21;22:354. doi: 10.1186/s12870-022-03731-5 (PMC9306182; doi:10.1186/s12870-022-03731-5)
Supplement: Supplementary file 1 — Additional file 1. [file 12870_2022_3731_MOESM1_ESM.docx]

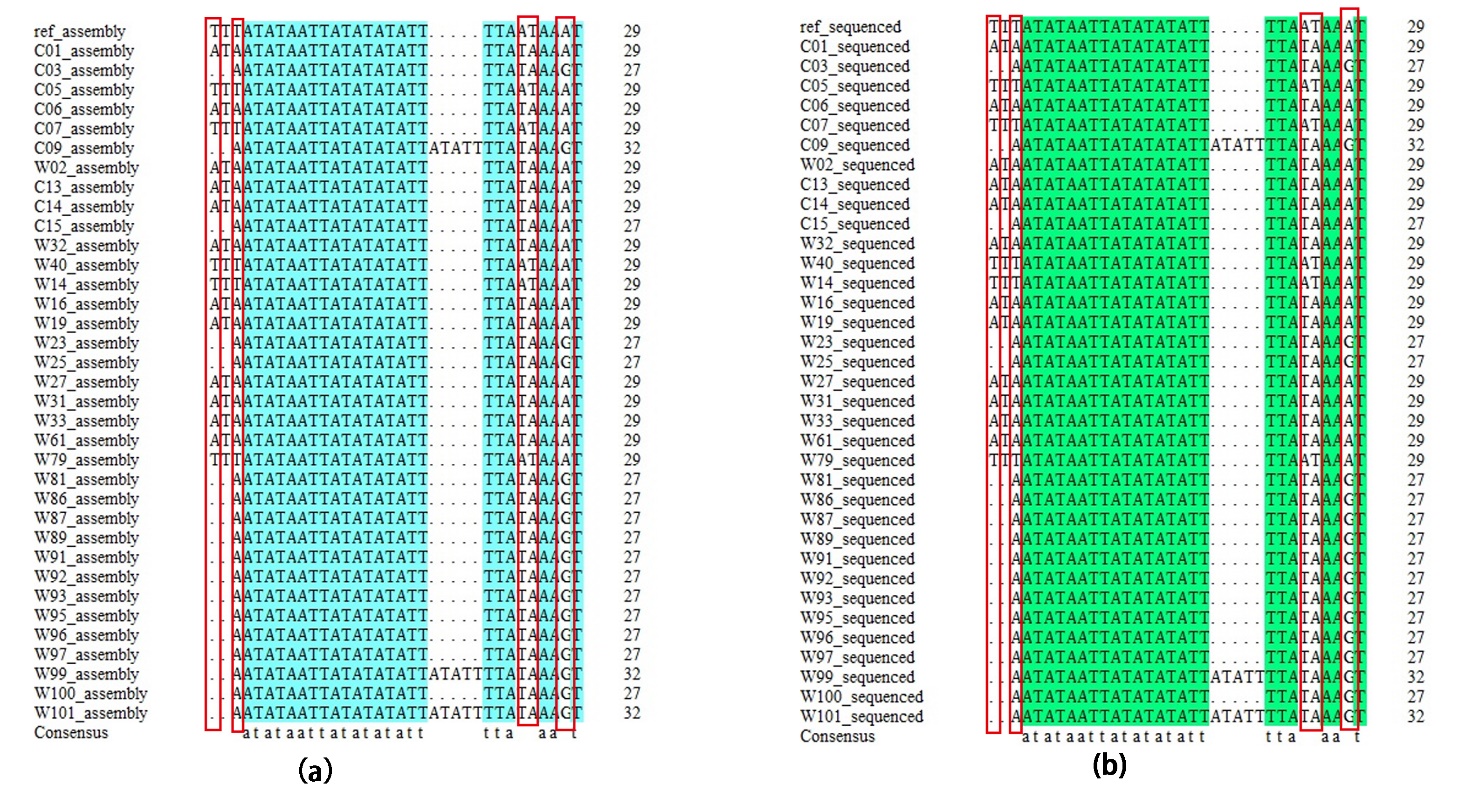


**Figure S1. The verification of SNP mutation sites of the first sequence by PCR.** (a) Detection of SNP by assembled sequences; (b) Verification of SNP by PCR. The red box indicates the location of the SNP.


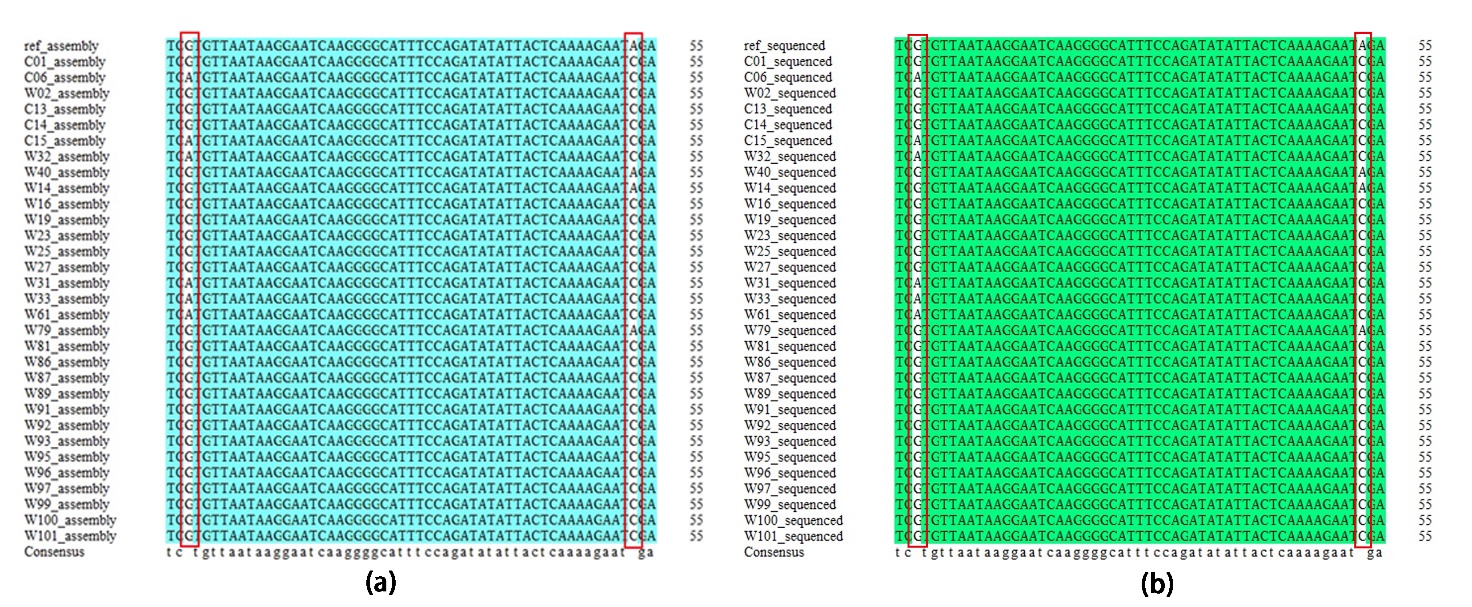


**Figure S2. The verification of SNP mutation sites of the second sequence by PCR.** (a) Detection of SNP by assembled sequences; (b) Verification of SNP by PCR. The red box indicates the location of the SNP.


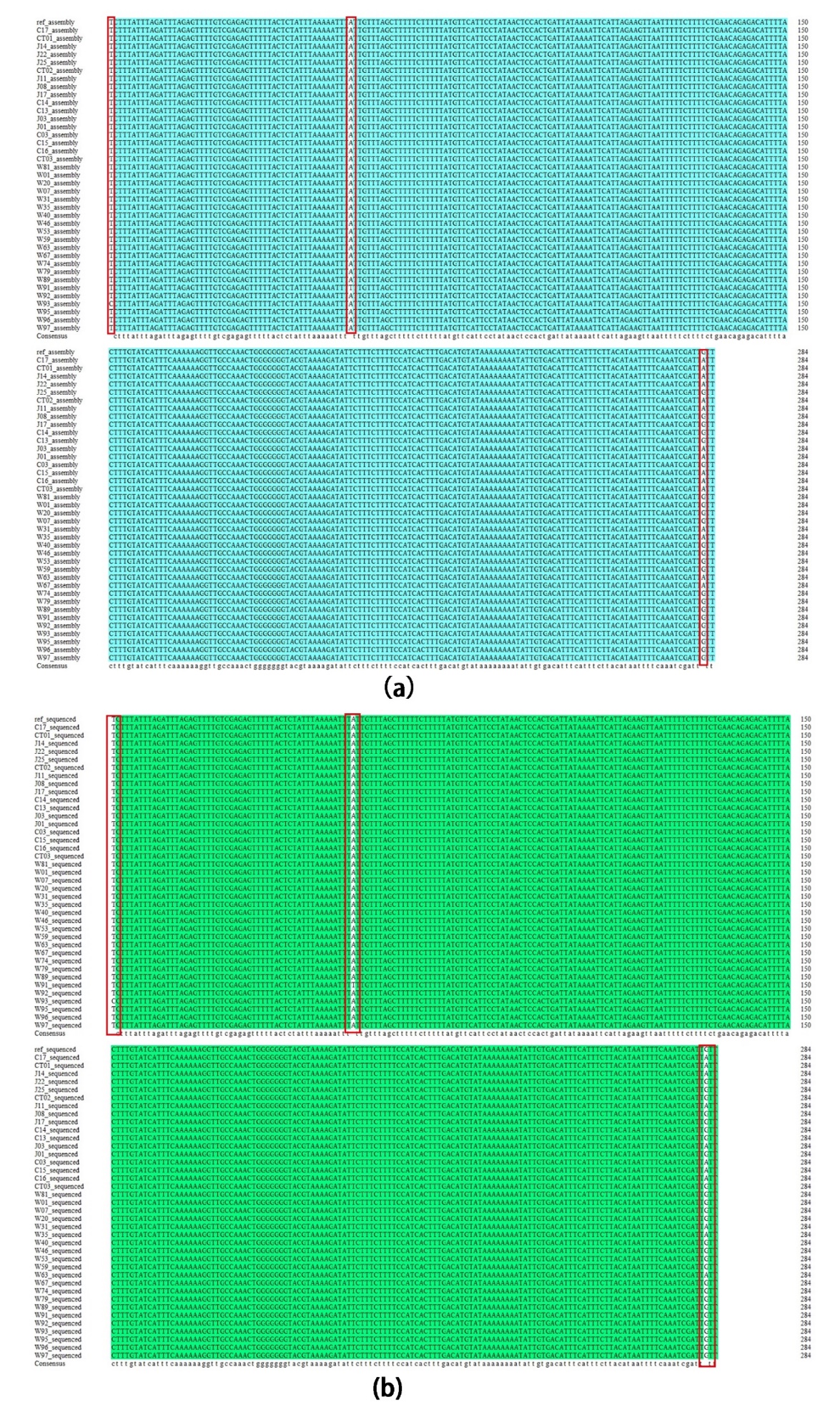


**Figure S3. The verification of SNP mutation sites of the third sequence by PCR.** (a) Detection of SNP by assembled sequences; (b) Verification of SNP by PCR. The red box indicates the location of the SNP.


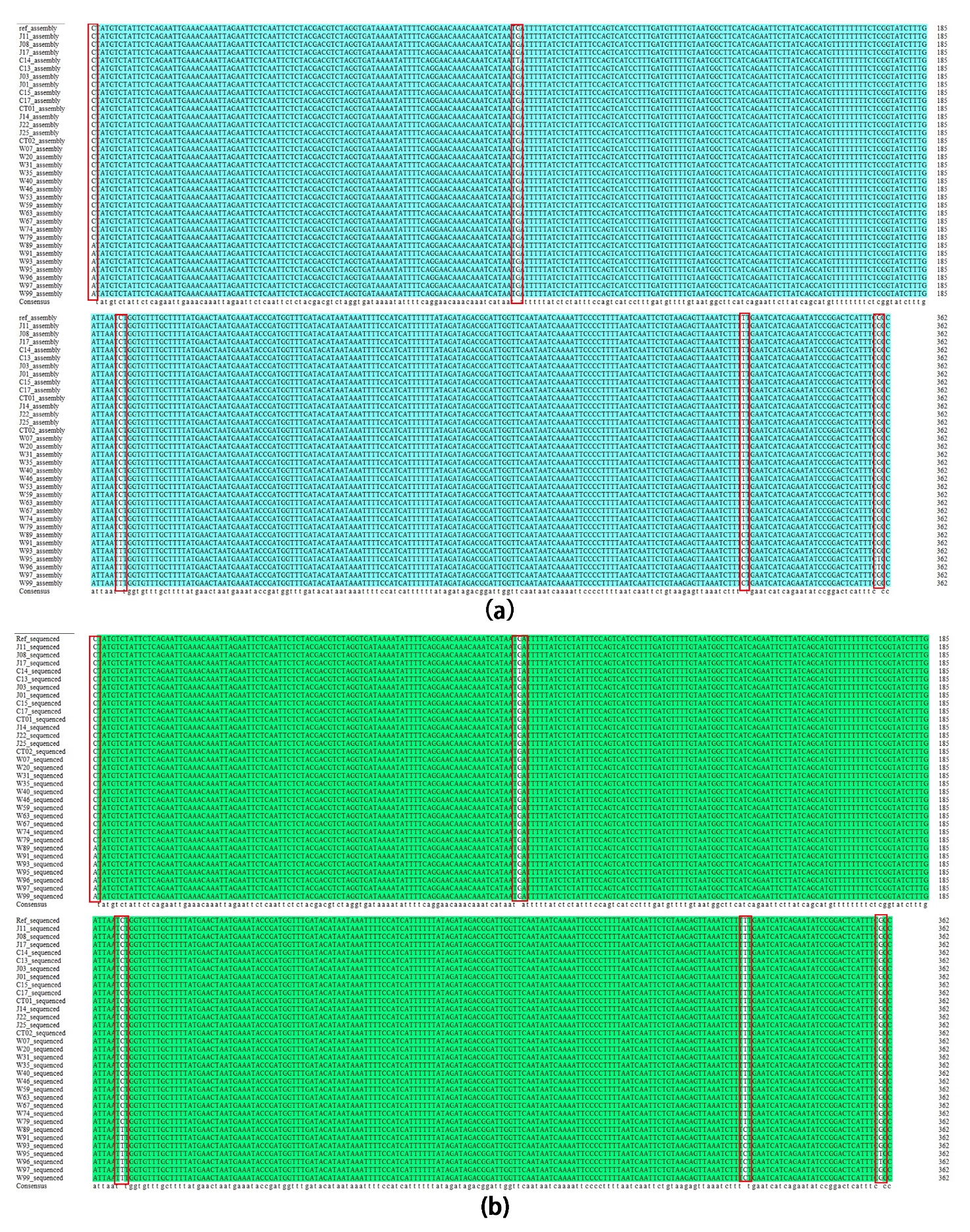


**Figure S4. The verification of SNP mutation sites of the fourth sequence by PCR.** (a) Detection of SNP by assembled sequences; (b) Verification of SNP by PCR. The red box indicates the location of the SNP.


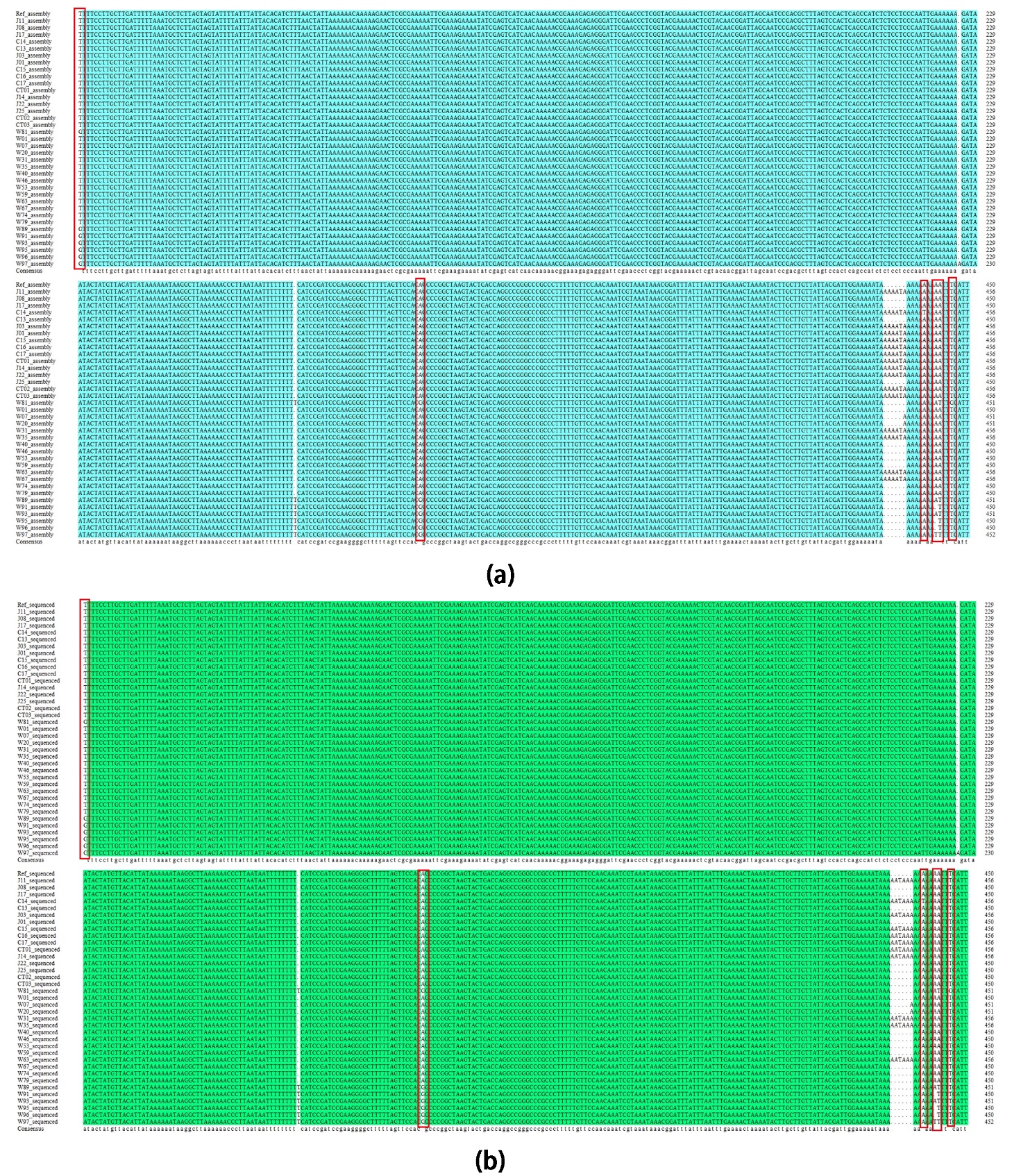


**Figure S5. The verification of SNP mutation sites of the fifth sequence by PCR.** (a) Detection of SNP by assembled sequences; (b) Verification of SNP by PCR. Red box indicates the location of the SNP.
